# The Effects of Oral *Rehmannia glutinosa* Polysaccharide Administration on Immune Responses, Antioxidant Activity and Resistance Against *Aeromonas hydrophila* in the Common Carp, *Cyprinus carpio* L

**DOI:** 10.3389/fimmu.2020.00904

**Published:** 2020-05-08

**Authors:** Jun-chang Feng, Zhong-liang Cai, Xuan-pu Zhang, Yong-yan Chen, Xu-lu Chang, Xian-feng Wang, Chao-bin Qin, Xiao Yan, Xiao Ma, Jian-xin Zhang, Guo-xing Nie

**Affiliations:** ^1^College of Fisheries, Henan Normal University, Xinxiang, China; ^2^Engineering Technology Research Center of Henan Province for Aquatic Animal Cultivation, Henan Normal University, Xinxiang, China; ^3^School of Life Science, Central China Normal University, Wuhan, China

**Keywords:** *Cyprinus carpio* L, polysaccharide, *Rehmannia glutinosa*, immunoregulatory, antioxidant, disease resistance

## Abstract

The effects of the oral administration of *Rehmannia glutinosa* polysaccharide (RGP-1) on the immunoregulatory properties, antioxidant activity, and resistance against *Aeromonas hydrophila* in *Cyprinus carpio* L. were investigated. The purified RGP-1 (250, 500, and 1,000 μg/mL) was co-cultured with the head kidney cells of the common carp. The proliferation and phagocytosis activities of the head kidney cells, and the concentration of nitric oxide (NO) and cytokines in the culture medium were determined. Next, 300 common carps (47.66 ± 0.43 g) were randomly divided into five groups; the two control groups (negative and positive) were administered sterile PBS and the three treatment groups were administered different concentrations of RGP-1 (250, 500, and 1,000 μg/mL) for seven days. Subsequently, the positive and treatment groups were infected with *A. hydrophila*, and the negative group was administered sterile PBS for 24 h. The concentration of NO, cytokines, lysozyme (LZM), and alkaline phosphatase (AKP) in serum, the total antioxidant capacity (T-AOC), the levels of malonaldehyde (MDA) and glutathione (GSH), and the total activities of superoxide dismutase (T-SOD), catalase (CAT), and glutathione peroxidase (GSH-Px) in the hepatopancreas of the common carp were tested. We observed that RGP-1 could significantly enhance the proliferation and phagocytosis activities (*P* < 0.05), besides inducing the production of NO, pro-inflammatory cytokines (TNF-α, IL-1β, IL-6, IL-12) and anti-inflammatory cytokines (IL-10, TGF-β) (*P* < 0.05) *in vitro*. The *in vivo* experimental results revealed that RGP-1 significantly enhanced NO production, protein levels of pro-inflammatory cytokines (TNF-α, IL-1β, IL-6, IL-12), LZM and AKP activities, and the antioxidant content (T-AOC, SOD, CAT, GSH, GSH-Px, and MDA) compared to that observed in the negative group prior to *A. hydrophila* infection (*P* < 0.05). NO, pro-inflammatory cytokines, LZM and AKP activities were significantly lower than that in the positive group after infection (*P* < 0.05). However, whether infected or not, the expression of anti-inflammatory cytokines (IL-10, TGF-β) increased significantly in the RGP-1-treated groups (*P* < 0.05). Therefore, the results suggested that RGP-1 could enhance the non-specific immunity, antioxidant activity and anti-*A. hydrophila* activity of the common carp, and could be used as a safe and effective feed additive in aquaculture.

## Introduction

*Cyprinus carpio* is the major commercial fish species in China. In recent years, the increased frequency of diseases in the breeding process of the common carp have severely hindered industrial development. According to statistics, in 2017, the economic loss resulting from diseases in China's fishery industry reached 3.405 billion yuan, which increased by 24.3% compared to that in 2016 (1). Therefore, it is of significant importance to determine effective antibiotic substitutes that can help improve the immunity and disease resistance of aquaculture animals, and construct a healthy aquaculture system with safe aquatic products based on the idea of “no antibiotics and no worries” in the aquaculture industry. In the strategy of discovering alternative antibiotics, based on the large numbers of research foundations and application prospects, traditional Chinese medicine (TCM) and polysaccharides used in TCM are ideal choices.

In recent years, increasing attention has been paid to the effect of TCM on immune regulation (2–6), and several studies have demonstrated that polysaccharides are one of the important active ingredients. For example, the polysaccharide AEPS derived from the roots of *Actinidia eriantha* could significantly increase the expression of cytokines in rat medullary dendritic cells (BMDCs) (7). *Smilax glabra Roxb* polysaccharide SGRP1 promoted the phagocytic activity of macrophages and increased the expression of NO and cytokines (8). In addition, polysaccharides CRP from *Collybia radicata* mushroom, TLH-3 from *Tricholoma lobayense*, and HEP-S from *Hericium erinaceus* fruiting bodies all significantly promoted the proliferation and phagocytosis of RAW264.7 cells, and induced the expression of NO and cytokines (9–11). Moreover, polysaccharides also play a crucial role in aquaculture. For instance, the addition of *Ganoderma lucidum* polysaccharide to the basic diet significantly improved the survival and growth rates and the digestive enzyme activities of *Macrobrachium rosenbergii* (12). Both *Ficus carica* polysaccharide and *Hericium caput-medusae* polysaccharide stimulated the immune response in grass carp, and upregulated the immune-related genes, thereby enhancing the host's disease resistance ability (3, 13). Polysaccharides from *Padina gymnospora* and *Astragalus* sp. also improved the non-specific and specific immune response in the immunity-related organs in the common carp, and enhanced the immune regulation and disease resistance of the host (14, 15). In addition, polysaccharides from algae and other species are also widely used in aquaculture (16, 17).

As a part of TCM, *Rehmannia glutinosa* is widely cultivated in the Henan, Shandong, Shanxi, and Shaanxi provinces. Studies have revealed that the *R. glutinosa* polysaccharide (RGP) is one of its major functional components. For example, RGP could improve the vascular inflammation in diabetic mice induced by streptozotocin, and exerted positive therapeutic effects on hyperglycaemia and hyperlipidaemia in a mice model (18). RGP could also induce the maturation of BMDCs (19), the proliferation of splenic lymphocytes and the IL-12 and IFN-γ expression regulated by T cells (20). In addition, RGP liposomes could enhance the antigen presenting ability of mature DC cells and increase the number of splenic lymphocytes, IgGs, central and effector memory cells *in vivo* (21). However, to the best of our knowledge, no research has been conducted on the immune regulation, antioxidant and disease resistance effects exerted by RGP on the common carp. Based on previous research, the objective of this study was to determine the immunomodulatory, antioxidant and disease resistance effects exerted by RGP-1 derived from *R. glutinosa*, and to explore its feasibility as an immunomodulatory agent.

## Materials and Methods

### *In vitro* Study

#### RGP-1 Isolation and Purification

The roots of *R. glutinosa* were collected from the Wuzhi County, Jiaozuo, China. The polysaccharides from the roots of *R. glutinosa* (RGP-1) were prepared and characterized, as previously described (22, 23). In brief, 100 g of the dried root powder of *R. glutinosa* were extracted to prepare the aqueous extract of the crude polysaccharide based on the following steps: 100°C (extraction temperature), 2 h (extraction time), 20.0 mL/g (ratio of water to raw material) and three extractions (number of extractions). The extraction solution was centrifuged and filtered through a 0.45 μm filter membrane. The filtrate was then concentrated to a 1 Lvolume by decompression at 70°C in a rotary evaporator. The aqueous fraction was added to four times volume of 95% ethyl alcohol to form a precipitate at 4°C overnight. The precipitate was isolated through centrifugation at 12,000 × *g* for 10 min at 25°C, and rinsed with absolute ethanol, acetone and diethyl ether twice in turn, and then lyophilized to obtain the crude polysaccharide with a yield of 12.6 g. The crude polysaccharide was dissolved in deionized water and centrifuged to remove the extra insoluble constituents, and 10 mL of the supernatant was introduced into a DEAE-cellulose 32 column (60 × 6 cm, Cl^−^ form), followed by stepwise elution with deionized water and 0.2 mol/L NaCl at a flow rate of 0.23 BV/h. The eluate was then collected and loaded onto a Sephacryl S-200HR column (2.6 × 100 cm), followed by elution with 0.2 mol/L NaCl at a flow rate of 0.32 BV/h. Finally, the eluate was collected, dialyzed with water, and lyophilized to obtain the pure RGP-1 with a yield of 3.1 g.

#### Head Kidney Cell Culture and Treatment

The head kidney cells were isolated from healthy common carps (24) and cultured in RPMI-1640 medium (GIBCO, USA) with 10% fetal bovine serum (GIBCO, USA), 100 unit/mL penicillin (Sigma, USA) and 100 μg/mL streptomycin (Sigma, USA) in 24-well tissue culture plates (Corning, USA; 2 × 10^6^ cells/mL). Following this, the cells were co-cultured with different concentrations of RGP-1 (0, 250, 500, 1,000 μg/mL) at 28°C for 24 h. The production of cytokines and NO in the culture supernatants were detected.

#### Head Kidney Cell Proliferation and Phagocytosis Assays

To determine the effect of RGP-1 on the proliferation of head kidney cells, RGP-1 (0, 250, 500, 1,000 μg/mL) was added to the 24-well tissue culture plates (2 × 10^6^ cells/mL). After incubating for 20 h, 20 μL of MTT (5 mg/mL) was added to each well, and the cells were re-incubated for another 4 h. The supernatant was then discarded and DMSO (150 μL) was added to each well. The absorbance values were measured at 570 nm using an EnSpire 2300 microplate reader (PE EnSpire, USA) (9, 25).

For phagocytosis, similar to the method mentioned previously, the supernatants were discarded after the cells were incubated with the RGP-1 (0, 250, 500, 1,000 μg/mL) for 20 h. One hundred microlitres of neutral red solution (0.1 %) was added to each well. The supernatants were then discarded after 4 h, and the cells were washed thrice with PBS to remove the neutral red. One hundred microlitres of a cell lysis buffer [ethanol: glacial acetic acid = 1:1 (v/v)] was added to each well, and the plates were incubated for 2 h to extract the phagocytosed neutral red. The absorbance values at 540 nm were measured using an EnSpire 2300 microplate reader (PE EnSpire) (9).

#### Nitrite Assay for the Estimation of NO

After a 24 h incubation period, the levels of NO in the culture medium were determined using the Total Nitric Oxide Assay Kit (Beyotime, Shanghai, China), according to the manufacturer's instructions. The absorbance values at 490 nm were determined using an EnSpire 2300 microplate reader (PE EnSpire). The NO concentrations were estimated by referring to a standard curve for a 5-fold serial dilution of NaNO_2_.

#### Enzyme-Linked Immunosorbent Assay (ELISA) for the Quantitative Analysis of Cytokines

The concentration of cytokines in the culture supernatant was determined using the ELISA with polyclonal antibodies prepared in our lab that evaluated the *in vitro* immune regulatory activity of RGP-1 in common carps (26). Briefly, 96-well plates (Corning, USA) were coated with culture supernatants in a coating buffer (0.05 M carbonate buffer, pH 9.6) and maintained overnight at 4°C. The plates were blocked with 10% skimmed milk powder and washed. The first antibodies (TNF-α, IL-1β, IL-6, IL-12, IL-10, and TGF-β, 1: 2000) were added to the plates, and incubated for 2 h at 37°C. Next, the plates were washed again, and the second antibody (goat anti-rabbit, conjugated with horseradish peroxidise, 1:10000) was added, followed by incubation for 1 h at 37°C and an additional wash. The chromogenic signals were developed at 37°C for 30 min using 3, 3′, 5, 5′-tetramethylbenzidine (TMB) as a substrate. The reactions were terminated using 50 μL of 2 N H_2_SO_4_, and the absorbance values at 450 nm were measured using a Microplate Reader (PerkinElmer, USA). The equivalent levels of TNF-α, IL-1β, IL-6, IL-12, IL-10, and TGF-β were determined by comparing to reference curves constructed using the corresponding standards. The results were expressed as the concentration of cytokines in the culture medium (pg/mL).

### *In vivo* Study

#### Experimental Design

To determine the effect of RGP-1 on immunity, antioxidants and disease resistance *in vivo*, we adopted the following experimental design (22). *Cyprinus carpio* L. were obtained from the “Aquaculture Breeding Base of Henan Aquatic Technology Extension Station,” Henan Province, China. Thereafter, the fish, which were normal feeding, disease-free, and non-injured, were subjected to routine physical and microbiological examination to confirm the absence of bacterial diseases or any abnormal clinical signs, and the 300 common carps (47.66 ± 0.43 g) were randomly divided into 15 300-L tanks (20 fish per tank). The fish were fed commercial diets twice daily to apparent satiation (10:00 and 17:00) (Table 1).

**Table 1:** Ingredients and proximate composition of the basal diet.

**Ingredients**	**Proximate composition (%)**		
Fish meal	20.0	Moisture	9.4
Soybean meal	19.0	Crude protein	33.6
Rapeseed meal	20	Crude lipid	6.8
Wheat bran	12	Crude fiber	8.3
Wheat flour	20	Ash	8.23
Soybean oil	2.0	Energy (MJ/kg)	16.1
Ca(H_2_PO_4_)_2_	1.5		
Salt	0.2		
Premix^*^	2.3		

* *Premix supplied the following minerals (g/kg) and vitamins (IU or mg/kg): CuSO_4_·5H_2_O, 2.0 g; FeSO_4_·7H_2_O, 25 g; ZnSO_4_·7H_2_O, 22 g; MnSO_4_·4H_2_O, 7 g; Na_2_SeO_3_, 0.04 g; KI, 0.026 g; CoCl_2_·6H_2_O, 0.1 g; Vitamin A, 900000IU; Vitamin D, 200000IU; Vitamin E, 4,500 mg; Vitamin K_3_, 220 mg; Vitamin B_1_, 320 mg; Vitamin B_2_, 1,090 mg; Vitamin B_5_, 2,000 mg; Vitamin B_6_, 500 mg; Vitamin B_12_, 1.6 mg; Vitamin C, 5,000 mg; Pantothenate, 1,000 mg; Folic acid, 165 mg; Choline, 60,000 mg*.

After 2 weeks of acclimation, the 15 tanks were divided into five groups, with three parallel subgroups for each treatment. Group 1 (G1, negative control) and 2 (G2, positive control) were administered 0.1 mL PBS; G3, G4, and G5 were administered 0.1 mL of different concentrations (250, 500, and 1,000 μg/mL) of RGP-1, respectively, once daily for 7 days. For sample collection, six fish per tank (except for the G2 tank) were randomly selected and anesthetized using ethyl 3-aminobenzoate (MS-222, 40 mg/L) after 7 days of the gavage trial. During the trial, ~30% of the culture water was replaced once a day with fresh, dechlorinated water at a similar temperature and the oxygen–saturation was maintained by aeration. The tanks were maintained under a natural light/dark regime. During the experimental period, the water quality parameters [mean ± standard deviation (SD)] monitored were as follows: water temperature, 26.0 ± 0.5°C; pH, 6.5–7.1, dissolved oxygen > 6.1 ± 0.5 mg/L; ${\text{NH}}_{4}^{+}$-N < 0.5 mg/L; NO_2_-N < 0.05 mg/L.

#### A. hydrophila Challenge Experiment

After the feeding trial, the fish remaining from each replicate (except in G1) were injected with 100 μL of *A. hydrophila* (Ah 01) (LD50 = 5 × 10^6^ CFU/mL) intraperitoneally, which was provided by our laboratory, and cultured as previously described (26). In the negative control group (G1), the fish were injected with 100 μL of sterilized PBS. The culture conditions were the same as previously mentioned. After 24 h of the infection, six fish were randomly selected per tank and anesthetized for the sample collection.

#### Sample Collection

After 7 days of gavage and 24 h of injection, six fish were anesthetized per tank using MS-222 (40 mg/L), and blood samples were collected from the caudal vein by puncturing with a syringe, and the serum was separated by centrifugation (4°C, 3,000 × *g*, 30 min). Finally, the fish were dissected to isolate the hepatopancreas. All samples were stored at −80°C until further analysis.

#### Nitrite Assay for the Estimation of NO

After 7 days of gavage and 24 h of injection, the NO levels in the serum were determined according to the method described in nitrite assay for the estimation of NO.

#### Antioxidant-Related Index Assay

After the 7 days gavage, the antioxidant stress ability of RGP-1 in common carps was measured. In brief, the tissue homogenate of hepatopancreas (10%) was prepared using sterile saline, the supernatant was collected after centrifugation (4°C, 8,000 × *g*, 10 min), and the total antioxidant capacity (T-AOC), malonaldehyde (MDA) and glutathione (GSH) levels and the total activities of superoxide dismutase (T-SOD), catalase (CAT), and glutathione peroxidase (GSH-Px) in the supernatant were determined according to the kit instructions (Nanjing Jiancheng Bioengineering Institute, Nanjing, China).

#### Immune-Related Index Assay

After the 7 days gavage and 24 h of injection, the levels of TNF-α, IL-1β, IL-6, IL-12, IL-10, and TGF-β in the serum were measured according to the method described in Enzyme-Linked Immunosorbent Assay (ELISA) for the quantitative analysis of cytokines. The serum lysozyme (LZM) and alkaline phosphatase (AKP) activities were measured according to the kit instructions (Nanjing Jiancheng Bioengineering Institute).

### Data Analysis

The SPSS statistical package version 25.0 (SPSS Inc., Chicago, IL, USA) was used for statistical analysis, and the normality and homogeneity of data were checked and confirmed. The data were reported as mean ± SD, and subjected to the one-way analysis of variance (ANOVA), followed by Duncan's multiple range test as a *post-hoc* test to evaluate the significant difference between treatments at *P* < 0.05.

## Results

### Effects of RGP-1 on the Proliferation and Phagocytosis of Head Kidney Cells

**Figure 1:**
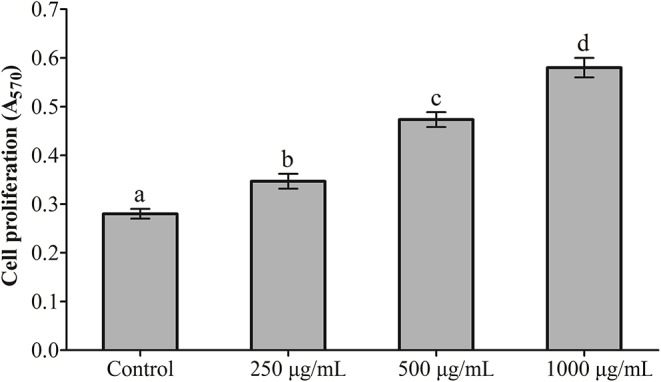
The effect of RGP-1 on the proliferation of the head kidney cells of common carps. The head kidney cells (2 × 10^6^ cells/well in 96-well plates) were incubated with the indicated concentrations of RGP-1 in DMEM at 28°C for 20 h; the proliferation activity of the head kidney cells was determined using an MTT kit. The data are expressed as the mean ± standard deviation (SD) (*n* = 6). The error bars represent the SDs; values marked with different letters are significantly different between the treatment groups (*P* < 0.05).

**Figure 2:**
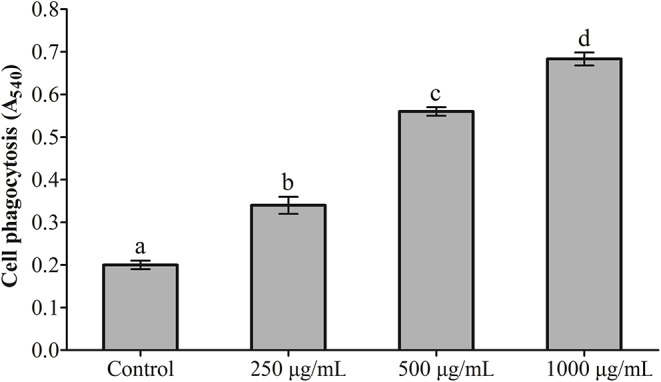
The effect of RGP-1 on the phagocytosis of the head kidney cells of common carps. The head kidney cells (2 × 10^6^ cells/well in 96-well plates) were incubated with the indicated concentrations of RGP-1 in DMEM at 28°C for 20 h; the phagocytosis activity of the head kidney cells was determined using the neutral red method. The data are expressed as the mean ± SD (*n* = 6). The error bars represent the SDs; values marked with different letters are significantly different between the treatment groups (*P* < 0.05).

The effects exerted by RGP-1 on the proliferation and phagocytosis of the head kidney cells are presented in Figures 1, 2. The proliferation and phagocytosis in the treatment groups were higher than that in the control group. As the RGP-1 concentration increased from 250 to 1,000 μg/mL, cell proliferation and phagocytosis increased gradually (*P* < 0.05). Particularly at 1,000 μg/mL, RGP-1 treatment increased cell proliferation and phagocytosis by 2.07 and 3.42 times, compared that in the control group (administered sterile PBS), respectively. In general, RGP-1 was non-toxic to the head kidney cells at the tested concentrations.

### Effects of RGP-1 on NO Production

**Figure 3:**
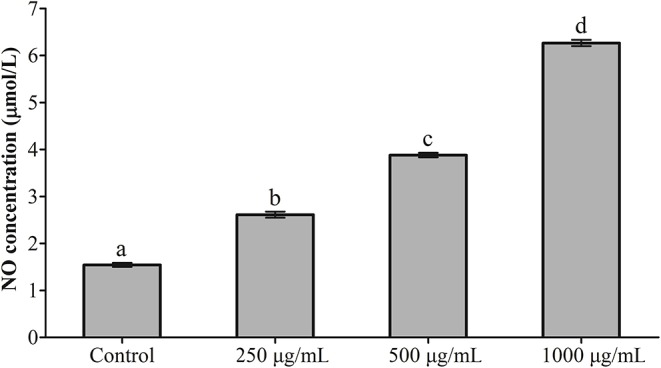
The effect of RGP-1-induced NO production in the head kidney cells of common carps. The head kidney cells (2 × 10^6^ cells/well in 96-well plates) were incubated with the indicated concentrations of RGP-1 in DMEM at 28°C for 24 h; the levels of NO in the culture medium were determined using the Total Nitric Oxide Assay Kit. The data are expressed as mean ± SD (*n* = 6). The error bars represent the SDs; values marked with different letters are significantly different between the treatment groups (*P* < 0.05).

The results of NO detection are presented in Figure 3 and Table 2. After exposure or gavage using different concentrations of RGP-1, the levels of NO in the head kidney cells and the serum of common carps in the groups that were only treated with RGP-1 increased in a dose-dependent manner both *in vitro* (Figure 3) and *in vivo* (Table 2), and were significantly higher than those in the control group (*P* < 0.05). The NO content in the serum of common carps increased significantly in the G2 group (only infected with *A. hydrophila*), whereas it reduced significantly in the groups pre-treated with RGP-1 after infection with *A. hydrophila* (*P* < 0.05, Table 2), and the ability of reducing NO production increased with the increase in RGP-1 concentration. Particularly, when the RGP-1 concentration was 1,000 μg/mL, the NO production reduced by 69.0%, and there was no significant difference with the control group (*P* > 0.05, Table 2).

**Table 2:** Effects of RGP-1 or/and *A. hydrophila* on NO levels, and the LZM and AKP activities in the serum of common carps.

**Treatments**	**NO (μmol/L)**	**LZM (μg/L)**	**AKP (U/mL)**
**Gavage treatment (7 days)**			
Control group	5.81 ± 0.27^a^	0.90 ± 0.03^a^	2.21 ± 0.05^a^
RGP-1 (250 μg/mL)	6.28 ± 0.26^a^	1.24 ± 0.05^b^	2.95 ± 0.04^b^
RGP-1 (500 μg/mL)	8.40 ± 0.35^b^	1.49 ± 0.02^c^	5.32 ± 0.09^d^
RGP-1 (1,000 μg/mL)	8.52 ± 0.53^b^	1.98 ± 0.03^d^	3.17 ± 0.06^c^
**Challenge treatment (24 h)**			
Negative control	5.72 ± 0.26^a^	0.92 ± 0.05^a^	2.26 ± 0.04^a^
Positive control	20.26 ± 0.26^d^	2.25 ± 0.09^c^	6.33 ± 0.06^c^
RGP-1 (250 μg/mL) + *A. hydrophila*	14.86 ± 0.70^c^	2.13 ± 0.05^c^	3.63 ± 0.23^b^
RGP-1 (500 μg/mL) + *A. hydrophila*	8.52 ± 0.15^b^	1.77 ± 0.14^b^	2.38 ± 0.21^a^
RGP-1 (1,000 μg/mL) + *A. hydrophila*	6.28 ± 0.15^a^	0.86 ± 0.03^a^	2.49 ± 0.17^a^

*Common carps (47.66 ± 0.43 g) were gavaged with indicated concentration of RGP-1 for 7 days, and then infected with A. hydrophila for 24 h, the levels of NO, and the activities of LZM and AKP in the serum were determined using the Total Nitric Oxide, Lysozyme and Alkaline Phosphatase assay kits, respectively. The data are expressed as the means ± SD (n = 6), and analyzed separately of the two sampling points. Values marked with different letters are significantly different between treatments (P < 0.05)*.

### Effects of RGP-1 on Antioxidant-Related Index

**Figure 4:**
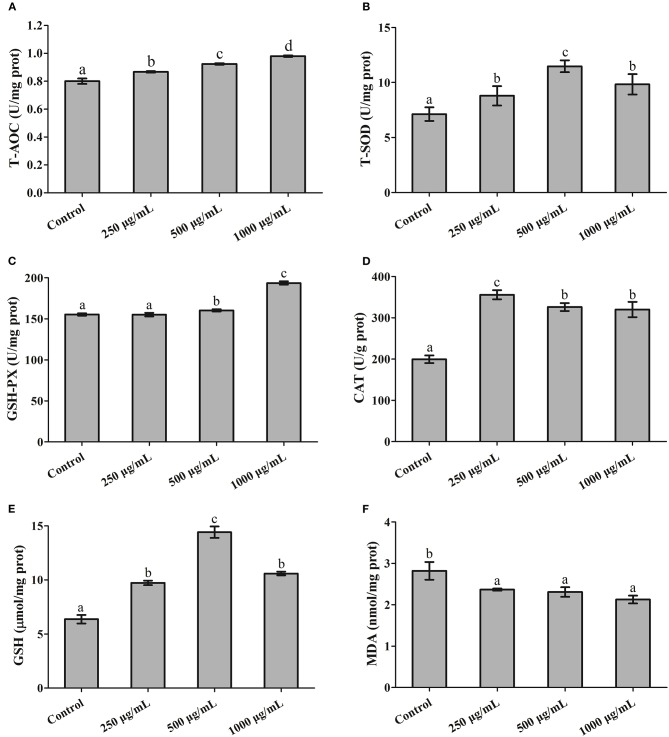
The effects of RGP-1 on the levels of antioxidant substances in the hepatopancreas of common carps. Common carps (47.66 ± 0.43 g) were gavaged with the indicated concentrations of RGP-1 for 7 days. **(A)** T-AOC, **(B)** T-SOD, and the levels of **(C)** GSH-Px, **(D)** CAT, **(E)** GSH, and **(F)** MDA in hepatopancreas were measured according to the kit instructions. The data are expressed as mean ± SD (*n* = 6). The error bars represent the SDs; values marked with different letters are significantly different between the treatment groups (*P* < 0.05).

**Figure 5:**
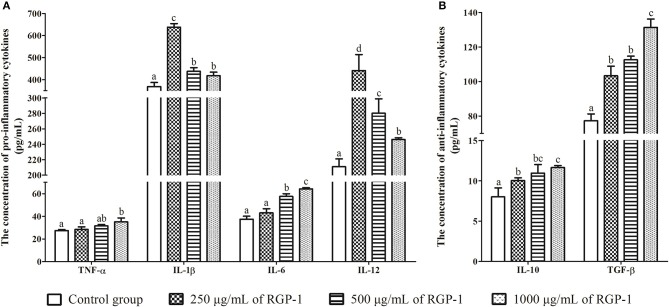
The effects of RGP-1 on the cytokine secretion from the head kidney cells. **(A)** The pro-inflammatory cytokines TNF-α, IL-1β, IL-6, and IL-12; **(B)** the anti-inflammatory cytokines IL-10 and TGF-β. Head kidney cells (2 × 10^6^ cells/well in 96-well plates) were incubated with the indicated concentrations of RGP-1 in DMEM at 28°C for 24 h; the cytokine levels in the culture medium were determined using the ELISA. The data are expressed as mean ± SD (*n* = 6). The error bars represent the SDs; values marked with different letters are significantly different between the treatment groups (*P* < 0.05).

As shown in Figure 4, at the end of the 7 days feeding experiment, the T-AOC and GSH-Px activity in hepatopancreas appeared to follow a dose-dependent pattern, and were significantly higher at a higher concentration (1,000 μg/mL of RGP-1) than in the control (*P* < 0.05, Figures 4A, 5C). The T-SOD activity and GSH levels were also higher in the treatment groups than in the control group (*P* < 0.05), and peaked at 500 μg/mL of RGP-1 (*P* < 0.05, Figures 4A, 5E). Similarly, the CAT activity peaked at the RGP-1 concentration of 250 μg/mL, and was significantly higher than that in the control group (*P* < 0.05, Figure 4D). Conversely, the MDA levels were significantly lower in the RGP-1 treatment groups, with the lowest activity recorded at 1,000 μg/mL of RGP-1 (*P* < 0.05, Figure 4F).

### Effects of RGP-1 on the Cytokine Secretion of Head Kidney Cells

The levels of inflammatory cytokines TNF-α, IL-1β, IL-6, IL-12, IL-10, and TGF-β in head kidney cells were used to evaluate the *in vitro* immunoregulatory activity of RGP-1. The results indicated that RGP-1 significantly stimulated the secretion of cytokines TNF-α, IL-6, IL-10, and TGF-β in a dose-dependent manner (Figure 5), and at 1,000 μg/mL concentration of RGP-1, the enhancement of TNF-α, IL-6, IL-10, and TGF-β production was ~1.28, 1.71, 1.45, and 1.70 times higher than that in the control group, respectively (*P* < 0.05). Meanwhile, the expression trend for IL-1β and IL-12 exhibited a “hump pattern” with increasing RGP-1 concentration. When the concentration of RGP-1 was 250 μg/mL, the expression levels of IL-1β and IL-12 were the highest, with the expression levels in the treatment groups higher than that in the control group (*P* < 0.05, Figure 5).

### Effects of RGP-1 on Serum Cytokine Secretion

Based on the *in vitro* experiment, we also determined the concentration of cytokines in the serum to further assess the immunoregulatory effects of RGP-1 *in vivo*. As shown in Table 3, RGP-1 significantly stimulated the secretion of cytokines TNF-α and IL-6 in a dose-dependent manner, whereas the expression trend for IL-1β and IL-12 exhibited a “hump pattern” with increasing RGP-1 concentration. However, the *in vivo* expression patterns of anti-inflammatory cytokines IL-10 and TGF-β were different from those *in vitro*. Compared to that in G1, RGP-1 could significantly increase the expression levels of IL-10 and TGF-β in the treatment groups (*P* < 0.05); however, there were no significant differences between the groups with different concentrations (*P* > 0.05).

**Table 3:** Effects of RGP-1 or/and *A. hydrophila* on cytokine secretion in the serum of common carps.

**Treatments**	**TNF-α (pg/mL)**	**IL-1β (pg/mL)**	**IL-6 (pg/mL)**	**IL-12 (pg/mL)**	**IL-10 (pg/mL)**	**TGF-β (pg/mL)**
**Gavage treatment (7 days)**						
Control group	24.12 ± 0.87^a^	365.28 ± 12.83^a^	33.42 ± 0.58^a^	168.85 ±5.61^a^	8.91 ± 1.68^a^	81.65 ± 1.57^a^
RGP-1 (250 μg/mL)	30.47 ± 3.21^b^	606.87 ± 15.83^c^	41.83 ± 1.93^b^	269.33 ± 5.68^d^	14.32 ± 1.37^b^	109.31 ± 2.78^b^
RGP-1 (500 μg/mL)	33.45 ± 1.05^b^	522.65 ± 14.54^b^	51.11 ± 0.64^c^	236.39 ± 4.10^c^	12.90 ± 2.48^b^	110.07 ± 0.66^b^
RGP-1 (1000 μg/mL)	37.98 ± 0.38^c^	513.06 ± 17.01^b^	60.49 ± 1.91^d^	213.97 ± 7.85^b^	12.63 ± 0.61^b^	112.76 ± 0.53^b^
**Challenge treatment (24 h)**						
Negative control	23.96 ± 0.99^a^	377.83 ± 16.25^a^	32.28 ± 0.75^a^	172.46 ± 6.15^a^	8.72 ± 2.59^a^	83.55 ± 2.64^a^
Positive control	37.10 ± 0.58^d^	636.29 ± 69.05^c^	68.83 ± 2.02^e^	263.41 ± 9.49^c^	12.39 ± 0.41^b^	109.19 ± 4.09^b^
RGP-1 (250 μg/mL) + *A. hydrophila*	37.32 ± 0.87^d^	495.44 ± 6.82^b^	52.07 ± 2.02^d^	235.27 ± 5.98^b^	16.52 ± 0.70^c^	108.98 ± 16.02^b^
RGP-1 (500 μg/mL) + *A. hydrophila*	33.31 ± 0.77^c^	487.23 ± 12.03^b^	47.87 ± 1.06^c^	169.56 ± 12.24^a^	10.08 ± 0.74^ab^	80.18 ± 3.31^a^
RGP-1 (1000 μg/mL) + *A. hydrophila*	27.74 ± 0.77^b^	466.61 ± 8.01^b^	41.90 ± 1.43^b^	169.72 ± 10.91^a^	9.83 ± 0.56^ab^	81.25 ± 16.86^a^

*Common carps (47.66 ± 0.43 g) were gavaged with indicated concentration of RGP-1 for 7 days, and then infected with A. hydrophila for 24 h, the cytokines levels in the serum were determined using ELISA. The data are expressed as the means ± SD (n = 6), and analyzed separately of the two sampling points. Values marked with different letters are significantly different between treatments (P < 0.05)*.

The results of cytokine detection in serum upon *A. hydrophila* infection are presented in Table 3. Compared with that in G1, the expression of pro-inflammatory cytokines TNF-α, IL-1β, IL-6, and IL-12 and anti-inflammatory cytokines IL-10 and TGF-β increased significantly in G2 (*P* < 0.05), which was only infected with *A. hydrophila*. Meanwhile, compared to G2, the expression of pro-inflammatory cytokines TNF-α, IL-1β, IL-6, and IL-12 in the RGP-1 treatment groups reduced significantly in a dose-dependent manner (*P* < 0.05). Particularly, for IL-12, the expression patterns in the RGP-1 (500 and 1,000 μg/mL) treatment groups and G1 had no significant difference (*P* > 0.05). With respect to the anti-inflammatory cytokines, RGP-1 significantly increased the IL-10 and TGF-β levels in the G3 group (250 μg/mL) (*P* < 0.05), while in the G4 and G5 groups (500 and 1,000 μg/mL), the expression levels of IL-10 and TGF-β reduced, and there were no significant differences with the levels in G1 (*P* > 0.05). In summary, it was observed that anti-inflammatory cytokines were more sensitive to RGP-1 (Table 3).

### Effects of RGP-1 on LZM and AKP Activities in the Serum

The LZM and AKP activities in the serum were also determined after RGP-1 feeding and *A. hydrophila* infection. As shown in Table 2, the activities of LZM and AKP could be significantly altered by RGP-1, and seemed to follow an upward trend (*P* < 0.05) with the increase in RGP-1 concentrations before infection. The LZM activity increased in a dose-dependent manner, while the AKP activity peaked at 500 μg/mL feeding. However, after infection, RGP-1 could affect the activities of LZM and AKP and reduce their functions significantly (*P* < 0.05), and these inhibitory effects followed a dose-dependent pattern. In particular, when the RGP-1 concentration was 1,000 μg/mL, the LZM and AKP activities in the treatment and negative control groups had no significant difference (*P* > 0.05).

## Discussion

Owing to their broad spectrum of therapeutic properties and relatively low toxicity, polysaccharides isolated from several species of fungi, algae, and fruits among others have attracted significant attention in biomedical research (9–11, 16, 27–29). In addition, along with the increasing emphasis on food safety, a large number of natural immunostimulants have been developed as feed additives or medicines to reduce the use of chemotherapeutic drugs in animal production (30), thereby reducing the development of drug-resistant bacteria, drug residues in organisms and other side effects caused by the excessive use of antibiotics (31). *R. glutinosa* polysaccharide (RGP) is a natural polysaccharide, which is the major active component in the Chinese herbal medicine species *R. glutinosa*, and it exhibited positive immune-enhancing and antioxidant properties in mammals, insects and fish by activating their non-specific and specific immune system (32–34). According to the reports, RGP treatment induced the maturation of natural killer (NK) cells and exerted an anticancer effect (35); RGP also could be used as a mucosal adjuvant for inducing the activation of immune responses in the lungs of mice (34). Furthermore, RGP could promote the activation of human dendritic cells by up-regulating co-stimulatory molecule expression and pro-inflammatory cytokine production (36). In addition, a dietary supplement of RGP effectively enhanced growth performance, increasing the activities of LZM, acid phosphatase (ACP), SOD, AKP and total protein (TP), up-regulating immune- (TNF-α, IL-1β, IL-8, IFN-γ) and growth-related (growth hormone, GH, insulin-like growth factor, IGF-I) genes, and reducing the mortality in *Luciobarbus capita* (33). However, reports on the application of RGP in common carp production are still limited. Moreover, immune regulation, antioxidant and anti-infection capacities against pathogens are often assessed while determining the effects of immunostimulants on the host (37–40). Therefore, the present study was conducted to investigate the effect of RGP-1 on the immunity, antioxidant capacity and disease resistance of common carps *in vitro* and *in vivo*.

As marker cells in the immune system that measure non-specific immune function, the proliferative and phagocytic activities of monocytes/macrophages are frequently used to determine the immunomodulatory effects of natural immunostimulants *in vitro* (9, 41). In this study, the proliferative and phagocytic activities of the head kidney cells increased with increasing dietary RGP-1 levels, and were higher than those in the negative control (Figures 1, 2). These results indicated that RGP-1 could improve the survival status of immune cells in the common carp and affect the non-specific immune function and response strength of the host (42), thereby guiding host inflammation and other immune processes (43, 44). Similar results were also observed in T cells (41, 45) and RAW264.7 cells (9, 46).

Macrophages are known to play a critical role in innate immunity, and attack, destroy and ingest foreign substances through the production of cytokines and NO (47), primarily because NO can activate the immune system, and can react with the superoxide anions produced by the respiratory burst of phagocytes to generate a strong oxidant-peroxynitrite (ONOOH), which can kill or inhibit the growth of several pathogenic microorganisms. Therefore, NO serves as a critical cellular defense factor in the anti-infective immunity of an organism (48, 49), and NO synthesis is an important mechanism underlying the non-specific immunity of macrophages (50). For teleosts, which rely on non-specific immune processes to enhance their immune response and resist pathogen infection (51), cytokines such as TNF-α, IL-1β, IL-6, IL-10, IL-12, and TGF-β, primarily produced by activated macrophages, also play an important role in the immune system. Once induced, they trigger a disease-resistant response of the immune system and help enhance the immune response. Therefore, NO and cytokines are frequently detected in immune responses, an observation being reported by an increasing number of immunologists (37, 52). For instance, dietary loquat leaf extract supplementation significantly upregulated immune-related genes in the intestine and improves innate immune responses (53). The Ziziphus jujube fruit extract ZJFE exhibited immune regulation potential in the skin mucosa of carps by regulating cytokine expression (54). In this study, RGP-1 treatment groups exhibited a significant increase in the production of NO and cytokines *in vitro* and *in vivo* (Figures 3, 5, Tables 2, 3). However, after infection with *A. hydrophila*, there was an effective reduction in the expression of NO and pro-inflammatory cytokines *in vivo* in the treatment groups (Tables 2, 3). Similar results were also observed for NO in mice (42), Japanese eel (*Anguilla japonica*) (55) and abalone (*Haliotis discus hannai Ino*) (56), and for cytokines in the common carp (22), yellow catfish (*Pelteobagrus fulvidraco*) (57), and mice (9, 46). These results suggested that RGP-1 could induce non-specific immunity by regulating the production of NO and cytokines, which promoted the immunity against pathogenic infection in common carps and improved their survival rate. However, some extracts significantly down-regulated the anti-inflammatory cytokines in the process of regulating the immune response of common carps (54), which may be attributed to the different types of extracts, dosage and treatment methods used. Furthermore, it has been reported that the common carp and *A. hydrophila* also contain NO. The proportion of this part NO in the entire test results and its effect on the results remain unknown; therefore, further studies are required to draw effective conclusions on this.

T-AOC, T-SOD, GSH-Px, and CAT are important antioxidant enzymes that form the first level of defense by preventing free radical formation (58), and their activities are critically connected (59). In addition, GSH and MDA are also commonly detected in antioxidant indexes (39, 60). For example, dietary Ferula (*Ferula asafoetida*) significantly increased antioxidant gene expression in the common carp (61), and Myrtle (*Myrtus communis* L., Myrtaceae) in zebrafish (62). Dose-dependent seaweed supplementation increased the plasma T-AOC as well as the GSH levels, and the CAT and SOD activities in the liver of the Atlantic salmon (*Salmo salar*) (63). *Allium mongolicum Regel* polysaccharide supplementation exerted significant protective effects against LPS challenge by preventing alterations in the SOD and GST levels in *Channa argus* (64). In the present study, RGP-1 treatment significantly enhanced the activities of T-SOD, CAT, and GSH-Px, increased the concentration of T-AOC and GSH, and reduced the concentration of MDA in the hepatopancreas (Figure 4). Similar results were also observed in mice (65, 66), suggesting that RGP-1 may reduce the levels of reactive oxygen free radicals and exert positive antioxidant effect on the common carp.

LZM and AKP are the key components in defense against pathogens and oxidative stress in species that lack of an adaptive immune system (67), and are the important innate immune parameters indicating the stress or disease condition (68, 69). Therefore, the LZM and AKP activities in serum were detected at the end of the experiment. Several immunostimulants (such as *Thymus vulgaris* essential oils, *Sargassum horneri*, and *Psidium guajava*) reportedly enhance the LZM and AKP levels in fish (70–72). For example, the inclusion of *Heracleum persicum* and Myrtle (*Myrtus communis* L., Myrtaceae) in the diet significantly elevated the LZM activity in the common carp (73) and zebrafish (62), respectively, and oral exopolysaccharide from *Lactococcus lactis* Z-2 significantly up-regulated the LZM and AKP activities in common carp (74). In this study, we also observed that the LZM and AKP activities in the serum of common carps fed with RGP-1 increased significantly and remained stable after infection with *A. hydrophila* (Table 2), which indicated that RGP-1, as an effective immune enhancer, could improve the LZM and AKP activities in the common carp.

In conclusion, RGP-1 exerts potent immunoregulatory effects at the cellular and host levels. It could improve the cellular proliferation and phagocytosis, besides inducing the release of cytokine and NO from cells, and could enhance the levels of antioxidant substances and the LZM and AKP activities in common carps after 7 days of gavage. Moreover, during the pathogen infection, RGP-1 also exerted anti-*A. hydrophila* effects *in vivo*. These results indicated that RGP-1 can be used as an immunostimulant for enhancing the immunity of common carps.

## Data Availability Statement

All datasets generated for this study are included in the article.

## Ethics Statement

This study conformed to the guidance of animal ethical treatment for the care and use of experimental animals, and was approved by the Institutional Animal Care and Use Committee of Henan Normal University. The fishes were anesthetized with diluted MS-222 before been euthanized, and all efforts were made to minimize suffering.

## Author Contributions

JF and GN conceived and designed the experiments, prepared the manuscript. JF, ZC, and YC performed the majority of the experiments, evaluated the data. XY, XZ, and XC participated in immune related index assay. XM, YC, and XW participated in antioxidant assay. JF, GN, JZ, and CQ revised the manuscript.

## Conflict of Interest

The authors declare that the research was conducted in the absence of any commercial or financial relationships that could be construed as a potential conflict of interest.
